# Clinical Considerations in the Selection of Preexposure Prophylaxis for HIV Prevention in Canada

**DOI:** 10.1155/2022/3913439

**Published:** 2022-08-30

**Authors:** David C. Knox, Robert Pilarski, Harvinder S. Dhunna, Amit Kaushal, Jonathan D. Adachi

**Affiliations:** ^1^Maple Leaf Medical Clinic, Toronto, ON, Canada; ^2^Clinique médicale La Licorne, Montreal, QC, Canada; ^3^Church Wellesley Health Pharmacy, Toronto, ON, Canada; ^4^Specialty Pharma Solutions, Oshawa, ON, Canada; ^5^University of Toronto, Toronto, ON, Canada; ^6^McMaster University, Hamilton, ON, Canada

## Abstract

According to the Public Health Agency of Canada, approximately 62,050 people were living with HIV in Canada in 2018, and of those, 13% were undiagnosed. Currently, no single strategy provides complete protection or is universally effective across all demographic groups at risk for HIV. However, HIV preexposure prophylaxis (PrEP) is the newest HIV prevention strategy that shows promise. To date, two products have received an indication for PrEP by Health Canada: emtricitabine/tenofovir disoproxil fumarate (Truvada®; FTC/TDF) and emtricitabine/tenofovir alafenamide (Descovy®; FTC/TAF). Despite the high efficacy of these PrEP intervention methods, access to PrEP in Canada remains low. Identifying and addressing barriers to PrEP access, especially in high-risk groups, are necessary to reduce HIV transmission in Canada. While guidelines published by the Center for Disease Control and Prevention (CDC) include FTC/TAF information, the efficacy of FTC/TAF for PrEP has not yet been considered in Canada's clinical practice guidelines. Thus, the current paper reviews data regarding the use of FTC/TDF and FTC/TAF for PrEP, which may be useful for Canadian healthcare providers when counseling and implementing HIV prevention methods. The authors highlight these data in relation to various at-risk populations and review ongoing clinical trials investigating novel PrEP agents. Overall, FTC/TDF PrEP is effective for many populations, including men who have sex with men, transgender women, heterosexuals with partners living with HIV, and people who use drugs. While there is fewer data reported on the efficacy of FTC/TAF to date, recent clinical trials have demonstrated noninferiority of FTC/TAF in comparison to FTC/TDF. Notably, as studies have shown that FTC/TAF maintains renal function and bone mineral density to a greater extent than FTC/TDF, FTC/TAF may be a safer option for patients experiencing renal and/or bone dysfunction, for those at risk of renal and bone complications, and for those who develop FTC/TDF-related adverse events.

## 1. Introduction

The Public Health Agency of Canada estimates that 62,050 people were living with HIV in Canada in 2018, and of those, 13% were undiagnosed [1]. Despite nearly four decades of public health prevention efforts, HIV incidence in Canada has remained stable, and 2,122 HIV cases were reported in 2019 (rate 5.6/100,000 per population) [2]. A 21% decrease in new HIV diagnoses was reported in 2020, which may be linked to lower demand for, and the ability to provide HIV testing during the COVID-19 pandemic [2]. Identifying and treating those living with HIV who remain undiagnosed and intensifying prevention efforts in demographic groups with the highest incidence of HIV infections are two approaches critical to ending the HIV epidemic in Canada.

HIV prevention methods historically have included HIV testing and linkage to care, HIV treatment as prevention (TasP), postexposure prophylaxis, screening and treatment of sexually transmitted infections (STI), access to condoms, substance use treatment programs, and harm reduction interventions. No single strategy provides complete protection or is universally effective across all demographic groups at risk for HIV.

Gay, bisexual, and other men who have sex with men (gbMSM) are consistently the highest risk group despite representing 3–4% of the adult male population in Canada [1]. In 2020, 71.4% of newly diagnosed cases were male, of which 60.8% reported male-to-male sexual contact, and 3.0% of those who reported male-to-male sexual contact also reported injection drug use [2].

HIV preexposure prophylaxis (PrEP) is the newest HIV prevention strategy. PrEP involves the use of antiretroviral medications in HIV-negative individuals to prevent new HIV infections. Multiple clinical trials have demonstrated the high efficacy of PrEP in preventing HIV infection in high-risk groups, with its effectiveness dependent on user adherence [3–7].

To date, two products have received an indication for PrEP by Health Canada: emtricitabine/tenofovir disoproxil fumarate (Truvada®; FTC/TDF) and emtricitabine/tenofovir alafenamide (Descovy®; FTC/TAF). *In vivo*, metabolites of FTC, TDF, and TAF inhibit the activity of HIV-1 reverse transcriptase by causing chain termination of nascent HIV DNA, thus disrupting the viral lifecycle and preventing incorporation of HIV DNA into the host genome, which is required to establish a chronic, lifelong HIV infection [8, 9]. TDF and TAF are both prodrugs of tenofovir (TFV), which undergoes phosphorylation to tenofovir-diphosphate (TFV-DP), the metabolite that is active against HIV. With TAF, plasma TFV levels are approximately 90% lower, while peripheral blood mononuclear cell (PBMC) concentrations of TFV-DP increase up to seven-fold compared to TDF [10, 11]. Increased plasma TFV levels are implicated in decreased renal function and bone density in both HIV treatment and prevention; these will be reviewed elsewhere in this paper [12, 13].

Despite this highly effective intervention, available data on uptake suggest that approximately 20–35% of individuals with indications for PrEP are accessing it [14, 15]. Identified barriers to PrEP uptake in Canada are numerous and include willingness to use PrEP and low perception of HIV risk among those demonstrating high risk on screening tools, such as the HIRI-MSM [16–19]. Additionally, PrEP uptake differs based on geography, secondary to varying provincial reimbursement programs and health care providers' comfort in prescribing PrEP [20–22]. Understanding the barriers to PrEP uptake in high-risk groups is essential in reducing the transmission of HIV in Canada. Health care providers play a critical role in educating patients on HIV risk and counseling on prevention methods. However, previous Canadian studies have demonstrated varying levels of knowledge about PrEP and comfort in prescribing PrEP [21, 22]. While a Canadian clinical practice guideline for PrEP was published in 2017, it preceded FTC/TAF receiving a Health Canada indication for PrEP [23]. Center for Disease Control and Prevention (CDC) PrEP guidelines have been updated to include FTC/TAF; however, the Canadian clinical practice guideline for PrEP has not been modified to reflect the available data on the efficacy of FTC/TAF for PrEP. This paper outlines important considerations for health care providers when choosing between FTC/TDF and FTC/TAF for PrEP as part of HIV prevention counseling and implementation.

## 2. Efficacy of FTC/TAF and FTC/TDF

### 2.1. FTC/TDF Efficacy across Different Trials

The efficacy of PrEP for HIV is strictly user-dependent, and adherence is the key to treatment success. The VOICE and FEM-PrEP trials are examples of poor efficacy due to low adherence [24–31]. Conversely, high adherence to PrEP translates to high efficacy. In trials such as iPrEX, its open-label extension (iPrEX OLE), and the Partners Demonstration Project, PrEP with FTC/TDF successfully prevented HIV infection in up to 100% of participants if taken at least four times a week [6, 32–34].

The effectiveness of oral daily PrEP versus event-driven (taken before and after sexual contact) PrEP is similar. The IPERGAY study, a randomized, multicentre, double-blind study that included gay men and transgender women, showed an 86% risk reduction with event-driven PrEP versus placebo in the double-blinded phase of the study with 400 participants and a 97% risk reduction in the open-label extension with 361 participants [35–37]. Despite the high protection rate, the sample size was too small to make a conclusion regarding the efficacy of event-driven PrEP. In the prospective cohort study ANRS PRÉVENIR, approximately half of the 3,067 participants chose to take event-driven PrEP, while the other half chose to use daily PrEP. According to preliminary results, six participants (three on daily, three on event-driven) became HIV-positive during the study period. While sexual behaviors differed between groups, the interim data suggest that participants are able to assess their own risk and, in conjunction with their HIV specialist, decide on which mode of PrEP administration best suits their current needs [38, 39]. Currently, prescribing event-driven PrEP is considered off-label as it has not been reviewed by any regulatory agency.

The risk of HIV infection is greater in people who inject drugs (PWID) in comparison to the population overall due to injections and sexual risk behaviors [40]. Only one randomized controlled trial evaluated the use of PrEP in this population [4], which demonstrated that TDF reduced the incidence of HIV by 49% compared with placebo. This study also showed a further risk reduction in participants who adhered to treatment compared with those who did not. The Canadian guidelines recommend that PrEP can be considered for use by PWID if they are at high risk for HIV.

### 2.2. Efficacy of FTC/TAF Compared with FTC/TDF in the DISCOVER Study

The DISCOVER study compared the association of emtricitabine with either tenofovir alafenamide (FTC/TAF) or tenofovir disoproxil fumarate (FTC/TDF) [12]. FTC/TAF demonstrated noninferiority to FTC/TDF at weeks 48, 96 (double-blinded phases), and 144 (open-label phase) of the trial. Pharmacokinetic (PK) data show that FTC/TAF penetrates PBMCs more rapidly than FTC/TDF, exhibits a four-to seven-fold higher intracellular concentration in comparison to FTC/TDF, and remains longer in the cells (16 days versus 10 days with FTC/TDF) when the medication is discontinued. These PK characteristics could allow a shorter lead-in period and result in increased efficacy at lower doses [41, 42].

Out of the 5,335 DISCOVER trial participants, 27 (0.5%) acquired HIV (11 in the FTC/TAF arm and 16 in the FTC/TDF arm). Five participants had suspected baseline infections, 19 had low levels of TFV-DP which was revealed via dried blood spot (DBS) analysis, and three patients had medium or high levels of FTC/TAF or FTC/TDF [41,43,44]. None of the participants had TDF-specific mutations. There was a delay between the DBS sample collection and the time of infection, suggesting that all participants who acquired HIV, including those with moderate to high levels of TDF, were infected due to nonadherence to PrEP.

Albeit highly effective, PrEP is not infallible. Rare cases of HIV transmission have been documented with sufficient levels of PrEP molecules. Mutations such as K65R, with or without M184V, Q151M, T69S insertion, and thymidine analog mutations (TAM) with M41L or L210W can render PrEP inefficacious [45–54].

## 3. Safety of FTC/TAF and FTC/TDF

The DISCOVER trial demonstrated that both FTC/TAF and FTC/TDF were well-tolerated for 96 weeks, and the rates of drug-related adverse events (AE) were similar between study arms (21% and 24%, respectively) [55]. Common AEs (>10%) were similar between groups and included STIs, diarrhea, nasopharyngitis, and upper respiratory tract infections. Serious AEs related to study drugs were rare at 0.1% in the FTC/TAF arm and 0.2% in the FTC/TDF arm. Adverse events leading to drug discontinuation were uncommon in both arms, at 1% for FTC/TAF and 2% for FTC/TDF.

FTC/TAF was superior to FTC/TDF in the DISCOVER trial for all prespecified bone mineral density (BMD) and renal biomarker safety endpoints [55]. At 96 weeks, there was a median body weight change of +1.7 kg in the FTC/TAF arm compared to +0.5 kg in the group receiving FTC/TDF. This is consistent with the known weight-suppressive effect of TDF and was also demonstrated in the iPrEX trial with a 1.7 kg weight gain observed in the placebo arm at 96 weeks versus a 0.6 kg weight gain in the FTC/TDF arm at week 72 [6]. In the open-label phase of DISCOVER, study participants who switched from FTC/TDF to FTC/TAF demonstrated an increase in weight (median change of +2.0 kg) consistent with the removal of the weight-suppressive effect of TDF [56]. The clinical significance of the observed weight gain has not been established, and further research is needed to understand the mechanisms responsible for the TDF-associated weight-suppressive effect.

At 96 weeks, both FTC/TDF and FTC/TAF were associated with reductions in HDL, LDL, and total cholesterol. These reductions were greater in the FTC/TDF arm secondary to the well-described lipid-lowering effect of TDF [55, 57]. Furthermore, the total cholesterol-to-HDL cholesterol ratio and fasting glucose levels were similar between both groups [55]. Upon entry to the DISCOVER trial, 4% of participants in the FTC/TAF arm were taking lipid-lowering therapy, and 2% initiated lipid-lowering therapy through week 144 [58].

## 4. Renal Considerations and Supporting Evidence

### 4.1. Risk Factors Associated with Renal AEs in PrEP Populations

Several risk factors can influence renal outcomes in PrEP populations. An age-associated decline in glomerular filtration rate (GFR) is observed in longitudinal and cross-sectional studies [59, 60]. Some populations are at an increased risk of accelerated kidney dysfunction due to concomitant medications [61–63], lifestyle (e.g., tobacco use, alcohol, and illicit substance use disorders) [64–66], comorbidities (e.g., hypertension, diabetes, established cardiovascular disease, as well as and family history) [61]. The presence of albuminuria, even with preserved estimated GFR (eGFR), is meaningful as it is known to increase the progression of renal disease. In a meta-analysis by Coresh et al., the authors reported an association across a range of cohorts between albuminuria and both end-stage kidney disease and mortality, with increasing degrees of urinary albumin excretion carrying a higher risk [67]. Further, the Kidney Failure Risk Equation, which is widely used to estimate renal risk, includes albuminuria as a variable, highlighting the implications of increased urinary protein excretion [68].

PrEP, specifically FTC/TDF, has been associated with renal function decline. Data from a retrospective observational analysis of risk factors for bone and renal diseases among new PrEP users showed that 15–37% of new PrEP users had risk factors for renal disease related to age, medication, or a diagnosis (including substance use disorder, alcoholism, and smoking) (Veradigm Health Insights (2015–2020), electronic health record database (unpublished raw data; Gilead data on file)). Similarly, the iPrEx OLE study reported a significantly greater decline in creatinine clearance (CrCl) for those starting PrEP at older ages, and baseline CrCl <90 mL/min predicted renal decline [69].

In comparison, FTC/TAF demonstrates a more favorable renal profile. A pooled analysis of 26 trials in people living with HIV demonstrated no cases of proximal tubulopathy in those taking TAF and a rate of drug discontinuation due to renal AEs in one-tenth of individuals on TDF [70].

These safety differences highlight the need to clearly characterize a patient's renal risk profile to inform PrEP therapy choice at initiation and follow-up.

### 4.2. Renal Safety in PrEP Studies

While there have been numerous reports and observational series describing renal dysfunction and proximal tubulopathy in the setting of antiretroviral therapy (ART) with TDF for HIV infection [71–75], the most compelling data for renal dysfunction related to TDF-based PrEP comes from a meta-analysis of studies involving PrEP alone, or in combination with FTC [76]. The authors identified 10 randomized trials that met the criteria and included a rise in creatinine as a defined renal AE. A total of 215 renal events were noted in the PrEP group and 137 events in the placebo group, with an odds ratio of 1.36 (95% CI, 1.09–1.71) for renal dysfunction and a number needed to harm of 167. While most renal events were noted to be grade 1 (creatinine 1.1–1.3 times the upper limit of normal), 54 events were classified as grade 2 or greater.

Prospective data confirm the risk for renal function associated with FTC/TDF. In the DISCOVER study, FTC/TAF was associated with the stability of renal function versus a decrease in eGFR in the FTC/TDF arm at the end of the 48-week treatment period [12]. This effect persisted up to the 96-week treatment period and for an additional 48 weeks in the open-label extension. Investigators monitored both the change in eGFR and evidence of proximal tubular dysfunction using *β*2-microglobulin-to-creatinine ratio and retinol binding protein-to-creatinine ratio. Participants on FTC/TDF with baseline levels of eGFR between 60 and 90 mL/min/1.73 m^2^ were found to exhibit numerically greater increases in tubular proteinuria compared to those who had baseline eGFR levels of 90 mL/min/1.73 m^2^ or higher [12]. This finding has important clinical implications, as up to 29% of PrEP users have mildly impaired renal function at baseline or have conditions that impact renal health, such as hyperlipidemia, hypertension, and diabetes [77, 78]. Renal AEs occurred in 10% of individuals in both groups, with a single case of Fanconi syndrome reported in the FTC/TDF arm of the trial. Looking at the renal safety profile of the two PrEP regimens within the DISCOVER trial, participants taking FTC/TDF who developed drug-related renal AEs had lower baseline eGFR and were more likely to be 50 years of age or older, an age group that is already at an increased risk for renal impairment. In this study, only 45% of participants with drug-related renal AEs had at least one of the examined renal risk factors [79].

As part of the iPrEx OLE study [69], changes in renal function were assessed in MSM and transgender women using daily PrEP with FTC/TDF. During a median of 72 weeks, there was a modest but statistically significant decline (−2.9%) in mean CrCl in study participants, with those starting PrEP at older ages (>40 years of age) showing a more pronounced effect. Additionally, those with marginal renal function at baseline (CrCl ≤90 mL/min/1.73 m^2^) had a higher probability of their CrCl falling to ≤ 60 mL/min or ≤ 70 mL/min while taking PrEP.

### 4.3. Clinical Implications

It is necessary to evaluate renal risk factors and renal function in individuals prior to PrEP initiation. For all individuals under consideration for PrEP treatment with either FTC/TDF or FTC/TAF, a baseline serum creatinine or eGFR test should be obtained, in addition to a urinalysis or urine albumin-to-creatinine ratio (ACR; Table 1). Follow-up monitoring should include quarterly measurement of creatinine/eGFR. Most clinicians include routine monitoring of urinalysis and/or ACR, which is more likely to detect proximal tubular dysfunction or albuminuria before any change in creatinine is noticed.

The required renal function values at baseline and follow-up differ between PrEP with FTC/TDF and FTC/TAF. For FTC/TDF, an eGFR ≥60 mL/min/1.73 m^2^ at initiation and follow-up is required. PrEP with FTC/TAF requires an eGFR ≥30 mL/min/1.73 m^2^ or ≤15 mL/min/1.73 m^2^ in those on hemodialysis. The eGFR should be measured within seven days. Additionally, FTC/TAF can be initiated before laboratory results are received, making this regimen better suited for rapid and same-day PrEP start, which may improve uptake and engagement in care.

## 5. Bone Considerations and Supporting Evidence

Populations at increased risk of BMD loss and osteoporosis include those who are over the age of 40 years, have vitamin D deficiency, are on medications that can affect bone health or have a disorder(s) associated with secondary osteoporosis, have long-term use of corticosteroids, and are transgender women [80, 81]. In certain populations at risk for HIV, some of these risk factors overlap. With the advent of new therapies and treatment strategies to prevent acquiring HIV, individuals may remain on these therapies for decades, and the development of chronic conditions such as osteoporosis and fractures is of greater concern.

In the context of PrEP, the DISCOVER study examined the effects of two different treatment strategies on BMD [12]. Secondary outcome measures included changes in the lumbar spine and hip BMD. Significant differences were observed between the groups, with i increasing BMD in the spine and hip observed in participants receiving FTC/TAF while declines in BMD were seen in those receiving FTC/TDF at 96 weeks, with significant differences of 2.6% and 1.4% in the spine and hip BMD, respectively [55]. Differences in fracture outcomes were not examined as the study was not powered to detect differences between the groups. These effects of PrEP on BMD are important, even in a younger population that may easily recover from fracture events because many individuals remain on PrEP for many years. Once peak BMD is established, typically by 30 years of age [61], it slowly declines throughout the remainder of the lifespan [82]. In individuals who are on PrEP between the ages of 20 and 30 years old, the medication may reduce bone accretion leading to a lower peak bone mass. If ongoing loss is seen, there may be significant consequences with a reduction in peak bone mass leading to skeletal fragility and increased incidence of fractures [83]. In a recent publication by Bouxsein et al., even small changes in BMD led to large reductions in fractures [84]. Indeed, Table 2 shows that a 2% change in total hip, femoral neck, or lumbar spine BMD leads to a 28% reduction in vertebral fracture, suggesting that even these small differences may result in a clinically meaningful reduction in fractures.

At present, no specific BMD screening is recommended before or during PrEP use, but bone health should be considered in PrEP candidates [61]. Vitamin D deficiency, if present, should be treated. For those at increased risk of fracture (e.g., prior fracture, age >65 years old, excessive alcohol consumption, low BMI, chronic corticosteroid use, or for those who are frail or sarcopenic), consideration for the type of PrEP therapy should be given, with greater consideration given to FTC/TAF over FTC/TDF. Studies of traditional treatment with bisphosphonates and denosumab have been shown to increase bone density in people living with HIV, although fracture outcomes are not available [84]. It would be reasonable to do a BMD measurement within three to six months in those commencing PrEP. Follow-up BMD measurement to monitor for the progression of bone loss may be done every 2–3 years at the discretion of the provider.

## 6. Practical Considerations When Prescribing Prep

### 6.1. Identifying Patients Who Might Benefit from PrEP

Identifying candidates for PrEP begins with a brief, targeted sexual and drug use history in all adults and adolescents. This should be a part of routine primary care but is often deferred due to lack of time or provider or patient discomfort [70]. Discussing sexual health should not be limited to specific patient groups, such as young, unmarried individuals, because data show that newly infected (HIV and STIs) individuals are adults and adolescents from all age groups, both sexes, and all genders, regardless of marital status [70]. Information about PrEP for the prevention of HIV infection should be provided to key groups (Table 3), as well as patients who do not report behaviors considered to be high risk for HIV when questioned but proceedingly request PrEP.

Patients might not disclose sexual or injection behaviors to their health care provider due to anticipated stigmatization. PrEP users are often stigmatized because of their association with HIV and are stereotyped as sexually irresponsible and promiscuous. Such prejudices negatively impact PrEP uptake and treatment adherence [85]. It is crucial that health care providers understand the role that stigma plays in discouraging and disrupting PrEP implementation, and they must intervene to overcome it.

### 6.2. Baseline Laboratory Investigations and Ongoing Monitoring

The Canadian clinical practice guideline for PrEP outlines baseline laboratory investigations and ongoing monitoring of PrEP users [23]. Baseline investigations include HIV testing, complete blood count, creatinine, urinalysis, pregnancy test (as appropriate), syphilis serology, hepatitis A, B, and C serology, and screening for gonorrhea and chlamydia via urine nucleic acid amplification tests (NAATs) and culture, or NAAT swabs at the appropriate anatomic sites depending on reported sexual activity (i.e., throat and rectum).

Follow-up HIV testing and creatinine are recommended 30 days after PrEP initiation to ensure PrEP was not started during an acute HIV infection and that there has been no decline in renal function [23]. Investigations every three months thereafter include HIV testing, creatinine, pregnancy test, and chlamydia and gonorrhea screening at appropriate anatomic sites. As per recommendations, yearly tests should be conducted for the hepatitis C antibody. In those who are nonresponders to the hepatitis B vaccine, the hepatitis B surface antigen should be tested yearly.

Because the components of both FTC/TAF and FTC/TDF have shown activity against hepatitis B virus (HBV), it is important to closely monitor the hepatic function of individuals with HBV infection for several months following PrEP discontinuation. PrEP withdrawal in patients with chronic HBV infection increases the risk of HBV flare. Therefore, consulting with a specialist experienced in treating HBV is recommended [23].

### 6.3. STIs and PrEP

Recent data show an increasing incidence of STIs amongst both non-PrEP and PrEP users, with the latter having a higher incidence of bacterial STIs and viral hepatitis C. This relationship is important to communicate with patients. PrEP, albeit effective against HIV, does not protect against any other STIs [86–90]. Baseline screening for STIs upon PrEP initiation should be repeated every three to six months in PrEP users, and HIV screening should be done every three months and upon diagnosis of an STI [23, 70].

### 6.4. Drug-Drug Interactions

Both TAF and TDF have minimal clinically significant drug-drug interactions because of the lack of CYP450 enzymatic metabolism [91]. They are substrates of P-glycoprotein, breast cancer resistance protein (BCRP), and multidrug resistance-associated protein 2 (MRP2) inhibitors. Drugs that affect P-glycoprotein and BCRP activity likely change the absorption of FTC/TAF. Owing to its intracellular metabolism, TAF is also more susceptible to clinically important drug interactions with P-glycoprotein manipulation [91]. Consequently, P-glycoprotein inducers decrease the absorption and plasma concentration of FTC/TAF, resulting in lower drug concentration. On the other hand, P-glycoprotein inhibitors increase the plasma concentration of FTC/TAF. Drugs that induce P-glycoprotein include anticonvulsants (e.g., carbamazepine, oxcarbazepine, phenobarbital, and phenytoin), antimycobacterial (rifabutin, rifampin, and rifapentine), and herbal products (St. John's wort, garlic). Coadministration of FTC/TAF with P-glycoprotein inducers is not recommended, with the exception of carbamazepine. In this case, the daily dose of FTC/TAF is increased to twice-daily administration [91].

Renal excretion is the main route of tenofovir elimination from the body [8, 9]. Therefore, coadministration of tenofovir with drugs that compete for tubular secretion may change the concentration of tenofovir and the competing drug, thereby increasing the risk of AEs. Some examples of such competitors include acyclovir, cidofovir, ganciclovir, valacyclovir, valganciclovir, aminoglycosides, and nonsteroidal anti-inflammatory drugs (NSAIDs) [92]. While this applies to both FTC/TAF and FTC/TDF, the latter carries a greater risk of nephrotoxicity due to higher plasma concentrations and a, consequentially, a higher concentration in renal tubular cells [91].

Neither TDF nor TAF is expected to interact with the following classes of medications: anticoagulant drugs, antiplatelet drugs, erectile dysfunction drugs, gastrointestinal agents (proton pump inhibitors, H2 inhibitors, anti-emetic agents), hormone replacement therapy/contraceptives/gender-affirming therapy, illicit/recreational drugs, opioid agonist therapy, lipid-lowering drugs, overactive bladder drugs, steroids, and antiparkinsonian drugs [9, 92]. For the list of established or potentially clinically significant drug interactions for FTC/TDF and FTC/TAF, health care practitioners should refer to their product monographs.

### 6.5. Pharmacokinetics and Its Clinical Implications

Tenofovir is a nucleotide analog of adenosine 5′-monophosphate that exists as a dianion at physiological pH. Its poor lipid membrane permeability results in low oral bioavailability. To overcome this PK limitation, tenofovir is available commercially in the form of prodrugs, such as TDF and TAF. In vivo, TDF and TAF are hydrolyzed to tenofovir, which is then phosphorylated to tenofovir diphosphate, the pharmacologically active metabolite. TAF and TDF have very different pharmacokinetics, which affect their safety and efficacy [8–11]. Some of the key PK parameters of TDF, TAF, and FTC are presented in Table 4.

In comparison with parent tenofovir, prodrug TDF has a better PK profile and antiviral activity *in vitro* and *in vivo* [93, 94]. The antiviral activity of TDF is 100-fold greater than that of parent tenofovir *in vitro* due to the rapid uptake of TDF and higher intracellular circulation of the active metabolite [94]. Also, oral TDF administration resulted in an approximate 8-fold increase in the PBMC exposure to the active metabolite in comparison to subcutaneous tenofovir [93].

**Figure 1:**
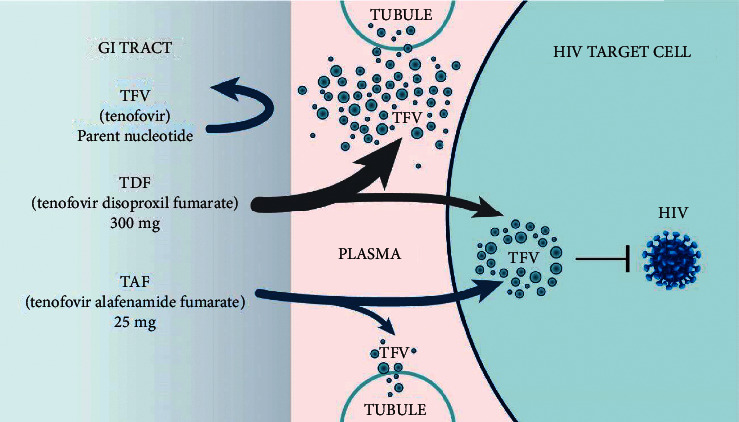
Higher TFV-DP levels in PBMCs with TAF vs TDF [95].

TAF is more efficient at loading TFV into PBMCs due to increased plasma stability (Figure 1). This results in plasma TFV levels that are approximately 90% lower than with TDF and a concentration of intracellular TFV-DP that is four-to seven-fold higher with TAF compared to TDF. Therefore, the TFV intracellular level, which is the best indicator of drug activity, is higher with TAF administration than TDF. In the iPrEx trial, drug concentrations in DBS were strongly associated with HIV incidence among those receiving PrEP [6, 33]. Drug concentrations associated with 90% protection were reached faster and lasted longer with TAF in comparison to TDF. Although both TDF and TAF produce high and therapeutically effective intracellular TFV-DP concentrations, TAF achieves this more quickly. The toxicities that are specifically related to high plasma TFV concentrations should not occur when using TAF.

### 6.6. Adherence to PrEP and Treatment Effectiveness

PrEP requires a high level of adherence to be effective [96]. While a total of 27 HIV infections were recorded in the DISCOVER trial, none of the participants acquired or developed tenofovir-resistant mutations [41, 43, 44]. A majority (19) of study participants who acquired HIV had insufficient drug levels of TDF or TAF. This finding is in accordance with previous studies, which showed that almost all seroconversions occur in individuals with suboptimal adherence [97] and very few are due to the transmission of TDF-resistant strains.

Accurately identifying and understanding the determinants of PrEP adherence can inform strategies to intervene with patients and maximize the benefit of PrEP. Factors that have been identified as contributors to suboptimal patient adherence include comorbidities (e.g., active substance abuse, mental health disorders, neurocognitive impairment), unstable housing and other psychosocial factors, missed clinic appointments, interruption of, or intermittent access to ARTs, cost and affordability of PrEP, low risk perception, and adverse drug effects [98, 99]. Multiple studies also described racial disparities in PrEP uptake and adherence [98]. Education, managing side effects, establishing routines, and providing reminder systems/tools are some strategies to support medication adherence.

### 6.7. Event-Driven PrEP

The results from the IPERGAY study demonstrated the effectiveness of event-driven FTC/TDF among high-risk MSM with frequent sex (median of 10 sex acts per month and eight partners every two months) [35–37]. Participants were instructed to take a loading dose of two pills of FTC/TDF or placebo with food 2 to 24 hours before sex, followed by a third pill 24 hours after the first drug intake and a fourth pill 24 hours later. The results showed an 86% (95% CI: 40–98, *p* = 0.002) reduction in the incidence of HIV with event-driven PrEP versus placebo. However, these results cannot be extrapolated to high-risk MSM with less frequent sexual activities and taking a more intermittent FTC/TDF regimen. At present, event-driven dosing of FTC/TDF is not recommended by regulatory authorities, and FTC/TAF has not been studied for event-driven PrEP. However, the PKs of TAF could make it a better candidate for event-driven PrEP, which warrants further investigation.

### 6.8. Same Day Start/Rapid Start

Initiating PrEP in a patient with undiagnosed HIV risks the development of virologic resistance to FTC and/or TDF/TAF, even though clinical trials with same-day PrEP initiation have shown that this is a rare occurrence [100]. It is important to establish HIV-negative status, ideally by a laboratory-based fourth-generation assay, prior to providing an initial or follow-up prescription for PrEP [23]. Same-day PrEP initiation following a review of the patient's medical history and a negative HIV point-of-care (POC) testing is generally safe and helps to retain patients in care [101, 102]. As access to POC HIV testing and laboratory services differ between health care settings in Canada, providers must balance the benefit of same-day PrEP initiation against the risks.

Same-day PrEP initiation is also an opportunity to link patients to care and reduce the amount of time spent at risk of acquiring HIV. While PrEP referrals from sexual health clinics are feasible, a large proportion of patients do not achieve PrEP initiation. In a study of patients referred to a PrEP clinic in an urban center, only 31% were ultimately linked to care [103]. When PrEP initiation is delayed (e.g., due to pending laboratory results), the risk of losing patients to follow-up and the time during which they may be exposed to HIV increase.

### 6.9. Access to PrEP

Access to PrEP in Canada varies significantly based on geography. Each provincial and territorial government offers a drug benefit plan for eligible groups. Most have specific programs for population groups that may require more enhanced coverage for high-cost medications. These groups include seniors, recipients of social assistance, and individuals with diseases or conditions that are associated with high-cost medications [104]. With a few exceptions for public coverage, PrEP may be prescribed by any knowledgeable prescriber licensed in their jurisdiction. At the time of writing, no Canadian province or territory listed FTC/TAF as a general benefit, and coverage is typically restricted to private payers (both individual and group insurance). Under exceptional circumstances, requests for drugs that are not listed in the formulary or for an indication that is not included in the special authorization criteria may be reviewed on a case-by-case basis. Health care providers should be aware of and leverage the assistance provided by patient support programs, which can provide copay assistance to cover the drug costs not paid by the individuals' insurance.

Sexual health clinics provide complete testing for individuals and refer them to PrEP prescribers. Patients can also access PrEP through an online consult with health care providers. Individuals may receive requisitions for laboratory investigations virtually and have their PrEP prescription sent to a pharmacy of their choosing. In addition, many pharmacies specializing in PrEP offer virtual counseling and medication delivery directly to patients.

Patient access to PrEP is influenced by health care provider familiarity prescribing PrEP. Maude et al.demonstrated that inadequate knowledge of PrEP and insufficient experience working with PrEP are among barriers to prescribing PrEP [105]. In Canada, referring patients to specialists for accessing PrEP also limits patient access due to lengthy wait times [106]. While PrEP increases the utilization of public health care resources [107, 108], the prevention of new HIV infections ultimately benefits public health and alleviates economic burden [109–112].

## 7. Discussion

PrEP is an innovative strategy in HIV prevention with benefits extending beyond physical health. Only a few studies reported on quality-of-life outcomes in PrEP users. An analysis of the impact of PrEP on health-related quality of life (HRQOL) of at-risk women and men living without HIV in the United States showed no significant difference with the general population, and HRQOL was maintained over time [113]. That PrEP administration does not negatively impact the quality of life is an important message for clinicians and at-risk individuals. Other domains of well-being, such as quality of sex life and perceived sexual pleasure, were investigated in a study by Van Dijk et al. PrEP users reported an increase in the quality of their sex lives, a decrease in their fear of HIV when having sex, and an increase in quality of life in general in the first six months of PrEP use [114]. These positive effects of PrEP should be clearly communicated through health care providers and awareness campaigns with the aim of reducing PrEP stigma and increasing PrEP uptake.

PrEP has been shown to be highly effective in adherent patients in the real-world setting [45]. Factors contributing to its efficacy are numerous and include potent antiretroviral activity against most HIV subtypes, early activity in the HIV reproductive cycle, high-resistance barrier with few cases of resistant strain transmission, and long intracellular half-life allowing high concentrations of medication in the PBMCs. Additionally, it is user-friendly with a convenient daily or event-driven administration. It has few drug—drug interactions and a well-established safety profile. Nonetheless, in order for PrEP to be efficacious, the user must commit to taking it consistently during high HIV-risk exposures [45]. PrEP adherence and engagement in care are the biggest challenges in the real-life setting and are directly related to its effectiveness [45, 115, 116].

Given FTC/TAF's safety profile and noninferiority compared with FTC/TDF, it should be considered for everyone at risk for HIV regardless of age and medical condition. When drug coverage between medications is equal, it should be the initial treatment for daily PrEP in those without a history of allergic or adverse reactions. Currently, FTC/TAF has not been evaluated and is not indicated in women and for event-driven PrEP [9]. FTC/TDF should still be used for women, in those who lack adequate coverage, and for event-driven use until more data are available. Due to potential drug-drug interactions, FTC/TAF and FTC/TDF PrEP users should be referred to experienced health care practitioners if tuberculosis treatment is to be initiated [117].

Among agents and modalities currently under investigation for PrEP, cabotegravir, a long-acting (LA) integrase inhibitor administered as an intramuscular injection, has been approved for PrEP by the U.S. Food and Drug Administration (FDA). The HPTN 083 trial, a randomized, double-blind, noninferiority study, showed the superiority of LA injectable cabotegravir over FTC/TDF in at-risk cisgender MSM and in at-risk transgender women who have sex with men [118, 119]. LA cabotegravir reduced the risk of HIV infection by 66% compared with FTC/TDF, and it maintained a significant advantage during an additional year of unblinded study after the three-year blinded phase of HPTN 083 [120]. Trial investigators reported seven breakthrough HIV infections throughout the entire study despite on-time LA cabotegravir administration. Researchers later established that using a sensitive RNA assay to screen for HIV in people using LA cabotegravir PrEP could unmask HIV infection earlier, reducing the risk of integrase inhibitor resistance [121]. In the HPTN 084 trial [120], LA cabotegravir was also superior to FTC/TDF for the prevention of HIV in HIV-uninfected women, and the number of seroconversions reported correlated with suboptimal oral PrEP adherence. Cabotegravir, unlike FTC/TDF or FTC/TAF, achieves prolonged drug exposure at therapeutic concentrations [121]. Consequently, an individual wanting to stop cabotegravir-based PrEP should continue oral PrEP for a year following the last injection to avoid HIV infection and the development of resistant strains of HIV.

Lenacapavir, a LA injectable capsid inhibitor administered subcutaneously every six months, is the first molecule of a novel class of HIV medications studied for HIV treatment and prevention [122]. It targets HIV at multiple stages of its replication cycle by binding directly between capsid protein subunits. Lenacapavir is highly potent compared to other ARTs. Thus, there is interest in its potential use in heavily treatment-experienced people living with HIV, as well as for PrEP [122]. Two PrEP trials, PURPOSE-1 and PURPOSE-2, will study HIV prevention relative to the background incidence rate with lenacapavir compared to FTC/TDF and FTC/TAF in cisgender women, cisgender MSM, transgender women and men, and gender nonbinary individuals [122]. These trials will also address the current data gap regarding FTC/TAF efficacy in cisgender women who are at risk of HIV acquisition via receptive vaginal intercourse [122].

## 8. Conclusion

There are robust data on the efficacy of FTC/TDF PrEP for the following populations: MSM, transgender women, heterosexuals whose partners are living with HIV, and PWID. In contrast, there is fewer efficacy data for FTC/TAF, which was approved by Health Canada in December of 2020. However, data from the DISCOVER trial demonstrated the noninferiority of FTC/TAF, a different prodrug with a more favorable PK profile, in comparison to FTC/TDF as once-daily PrEP in MSM and transgender women who have sex with men. As reviewed above, FTC/TAF compared to FTC/TDF was associated with improved maintenance of renal function and BMD. Therefore, FTC/TAF may be a safer option, specifically for those who have renal and/or bone dysfunction, who are at risk of declining renal and/or bone health, and who develop FTC/TDF-related AEs.

## Figures and Tables

**Table 1:** Laboratory assessments [80, 123–126].

Assay type	Baseline	Every three months	Every six months	Every 12 months
Serum creatinine	X	X		
eGFR	X	X		
eCrCL	X		X^a^	X^b^
ACR	X	^X^		

^a^Patients over the age of 50 or those who have an estimated CrCl (eCrCl) less than 90 mL/min/1.73 m^2^ at initiation. ^b^All other daily oral PrEP patients.

**Table 2:** Estimated fracture risk reduction associated with BMD improvement [83].

	Vertebral fracture (%)	Hip fracture (%)	Non-vertebral fracture (%)
*Δ in total hip BMD*			
2%	28	16	10
4%	51	29	16
6%	66	40	21

*Δ in femoral neck BMD*			
2%	28	15	11
4%	55	32	19
6%	72	46	27

*Δ in lumbar spine BMD*			
2%	28	22	11
8%	62	38	21
14%	79	51	30

BMD, bone mineral density.

**Table 3:** People living without HIV that may benefit from PrEP [23, 70].

(i) MSM or transgender women who engage in unprotected anal sex, particularly receptive anal sex
(ii) MSM or transgender women with multiple anal sex partners
(iii) MSM or transgender women with syphilis or rectal STIs
(iv) Individuals with one or more HIV-positive sex partners who have detectable viral loads or are not taking ART
(v) Individuals who have been prescribed one or more courses of nonoccupational postexposure prophylaxis (nPEP) with ongoing high-risk behavior
(vi) Serodiscordant couples who want a safer conception strategy
(vii) Injection drug users
(viii) Commercial sex workers or individuals who engage in transactional sex
(ix) Individuals who use stimulant drugs, such as methamphetamine, while engaging in high-risk sexual behaviors
(x) Individuals who request PrEP

**Table 4:** Pharmacokinetics parameters of TDF, TAF, and FTC.

	TDF	TAF	FTC
Absorption	(i) Plasma half-life: ∼0.4 minutes	(i) Plasma half-life: ∼30 minutes	(i) Plasma half-life: ∼10 hours
	(ii) Bioavailability: ∼25% (fasting)	(ii) Bioavailability: not reported	(ii) Bioavailability: 93% (hard capsule)

Distribution	Transported by renal transport proteins hOAT 1 and 3, and MRP4.	Transported by P-glycoprotein, BCRP, OATP1B1, and OATP1B3.	Emtricitabine is a substrate of MATE1 but not of OCT1, OCT2, P-glycoprotein, BCRP or MRP2 transporters.
		Unlike tenofovir, TAF is not a substrate for renal transporters OAT1 and OAT3.	

Metabolism	No P450 involvement	Minimal CYP3A4 metabolism	Undergoes minimal biotransformation via oxidation and glucuronide conjugation

Excretion	32 ± 10% renally excreted unchanged	<1% renally excreted unchanged	∼86% renally excreted (13% as metabolites)
	Tenofovir is renally eliminated by both glomerular filtration and active tubular secretion.	Tenofovir is renally eliminated by both glomerular filtration and active tubular secretion.	

BCRP, breast cancer resistance protein; FTC, emtricitabine; hOAT, human renal organic anion transporter; MRP2, multidrug resistance-associated protein; OAT, organic anion transporter; OATP, organic anion-transporting polypeptides; OCT, organic cation transporter; TAF, tenofovir alafenamide; TDF, tenofovir disoproxil fumarate.
